# Using FLIM-FRET to Measure Conformational Changes of Transglutaminase Type 2 in Live Cells

**DOI:** 10.1371/journal.pone.0044159

**Published:** 2012-08-31

**Authors:** Nicholas S. Caron, Lise N. Munsie, Jeffrey W. Keillor, Ray Truant

**Affiliations:** 1 Department of Biochemistry and Biomedical Sciences, McMaster University, Hamilton, Ontario, Canada; 2 Department of Chemistry, University of Ottawa, Ottawa, Ontario, Canada; University of Iowa, United States of America

## Abstract

Transglutaminase type 2 (TG2) is a ubiquitously expressed member of the transglutaminase family, capable of mediating a transamidation reaction between a variety of protein substrates. TG2 also has a unique role as a G-protein with GTPase activity. In response to GDP/GTP binding and increases in intracellular calcium levels, TG2 can undergo a large conformational change that reciprocally modulates the enzymatic activities of TG2. We have generated a TG2 biosensor that allows for quantitative assessment of TG2 conformational changes in live cells using Förster resonance energy transfer (FRET), as measured by fluorescence lifetime imaging microscopy (FLIM). Quantifying FRET efficiency with this biosensor provides a robust assay to quickly measure the effects of cell stress, changes in calcium levels, point mutations and chemical inhibitors on the conformation and localization of TG2 in living cells. The TG2 FRET biosensor was validated using established TG2 conformational point mutants, as well as cell stress events known to elevate intracellular calcium levels. We demonstrate in live cells that inhibitors of TG2 transamidation activity can differentially influence the conformation of the enzyme. The irreversible inhibitor of TG2, NC9, forces the enzyme into an open conformation, whereas the reversible inhibitor CP4d traps TG2 in the closed conformation. Thus, this biosensor provides new mechanistic insights into the action of two TG2 inhibitors and defines two new classes based on ability to alter TG2 conformation in addition to inhibiting transamidation activity. Future applications of this biosensor could be to discover small molecules that specifically alter TG2 conformation to affect GDP/GTP or calcium binding.

## Introduction

Transglutaminase type 2 (TG2; EC 2.3.2.13) is a multi-functional enzyme capable of catalyzing several calcium-dependent reactions, including a transamidation reaction (covalent cross-link) between the γ-carboxamide group of a peptide bound glutamine and a variety of amine substrates [1], in both an intra- and extracellular context [2]. Alternatively, TG2 can also hydrolyze GTP, where it acts as a G-protein mediating the phospholipase C signalling cascade [3], [4]. These cellular roles of TG2 are reciprocally regulated by a large conformational change [5], [6]. Crystal structures of TG2 have been solved indicating that GDP/GTP bound TG2 adopts a ‘closed’ conformation that is catalytically inactive as a cross-linking enzyme [6]. Conversely, an additional crystal structure shows that a substrate-mimicking peptide inhibitor bound to TG2 extends the enzyme to an ‘open’ conformation [6]. This suggests that the open conformation represents the enzymatically active version of the enzyme, yet to date no crystal has been solved with both calcium ions and a substrate bound to TG2. Under normal physiological conditions, intracellular calcium levels are low and the majority of the TG2 population is bound with guanosine nucleotides in a closed conformation [7]. However, under specific cell stress conditions, calcium levels rise dramatically causing a shift in the TG2 population towards its open and enzymatically active cross-linking conformation.

Despite the breadth of information that can be extracted from generating crystal structures, this work is time-consuming and assumes that the purified protein that packs into crystal arrays *in vitro* is representative of the protein conformation *in vivo*. As an alternative method, we generated a triple fusion of human TG2 between two fluorescent probes in order to measure conformational differences between the enzymatic roles of TG2 in live cells using Förster resonance energy transfer (FRET). FRET is a well-established method of measuring molecular interactions as well as conformational changes in live cells. FRET involves the non-radiative transfer of energy between a donor and an acceptor molecule [8]. FRET requires that the donor and the acceptor molecule be within a close spatial proximity of each other, where the Förster radius of a FRET pair refers to the distance at which the efficiency of energy transfer is 50% (∼8 nanometres for mCer and eYFP) [8]–[10]. This 8 nm spacing represents the distance between the fluorophore cores, yet when the outer valence shell diameters of the fluorescent proteins are considered, this proximity essentially represents a direct protein-protein interaction. Another requirement of FRET is a high degree of overlap between the emission spectrum of the donor molecule and the excitation spectrum of the acceptor molecule. The most quantitative method of measuring FRET in live cells is by using fluorescent lifetime imaging microscopy (FLIM) [11]. Fluorescence lifetime refers to the period of time a fluorescent molecule stays in an excited state prior to returning to ground state and emitting a photon [12]. The lifetime of a fluorophore can be affected by the biochemical and biophysical properties of its microenvironment, where FRET leads to a decrease in the fluorescence lifetime of the donor molecule that can accurately be measured [13]. Contrary to other spectral methods of measuring FRET, such as sensitized emission FRET (SE-FRET) [14], the ability to measure fluorescence lifetime of fluorescent proteins expressed in live cells is less dependent of relative probe concentrations and intensities, photo-bleaching as well as spectral bleed through [13], [15]. The accuracy of FRET measurement is further enhanced by this genetically-encoded triple fusion that insures a relative donor-acceptor fluorophore concentration of 1∶1. To measure lifetimes in the time-domain (TD-FLIM), the fluorescent samples are excited with a femtosecond pulsing laser coupled to a time-correlated single photon counting (TCSPC) system [16]. Using FLIM to measure FRET, we have generated a biosensor that provides a novel tool to quantitatively measure TG2 conformations in a live cell context. This sensor provides an assay to quickly measure the effects of cell stresses, point mutations and chemical inhibitors on the conformation of TG2. Using this assay, we have uncovered new mechanistic insights into the activity of TG2 inhibitors NC9 and Cp4d and have established a new way to classify TG2 inhibitors based on their ability to affect the conformation of the protein.

TG2 has been linked to a myriad of human disorders including: inflammatory, autoimmune and neurodegenerative diseases, yet the pathological role of TG2 in these conditions remains elusive. Much of the early work done on TG2 has focused on its aberrant cross-linking of substrates as being the cause of pathology; however growing evidence suggests that the subcellular localization and the conformation of TG2 are also critical in the regulation of cell death processes. Therefore, we believe that examining TG2 *in vivo* is critical to improving our understanding of TG2 and its role in multiple disease pathologies. This biosensor provides a universal tool capable of rapidly assessing the conformations of TG2 while providing additional information about the subcellular localization of TG2 in live cells.

## Results

**Figure 1:**
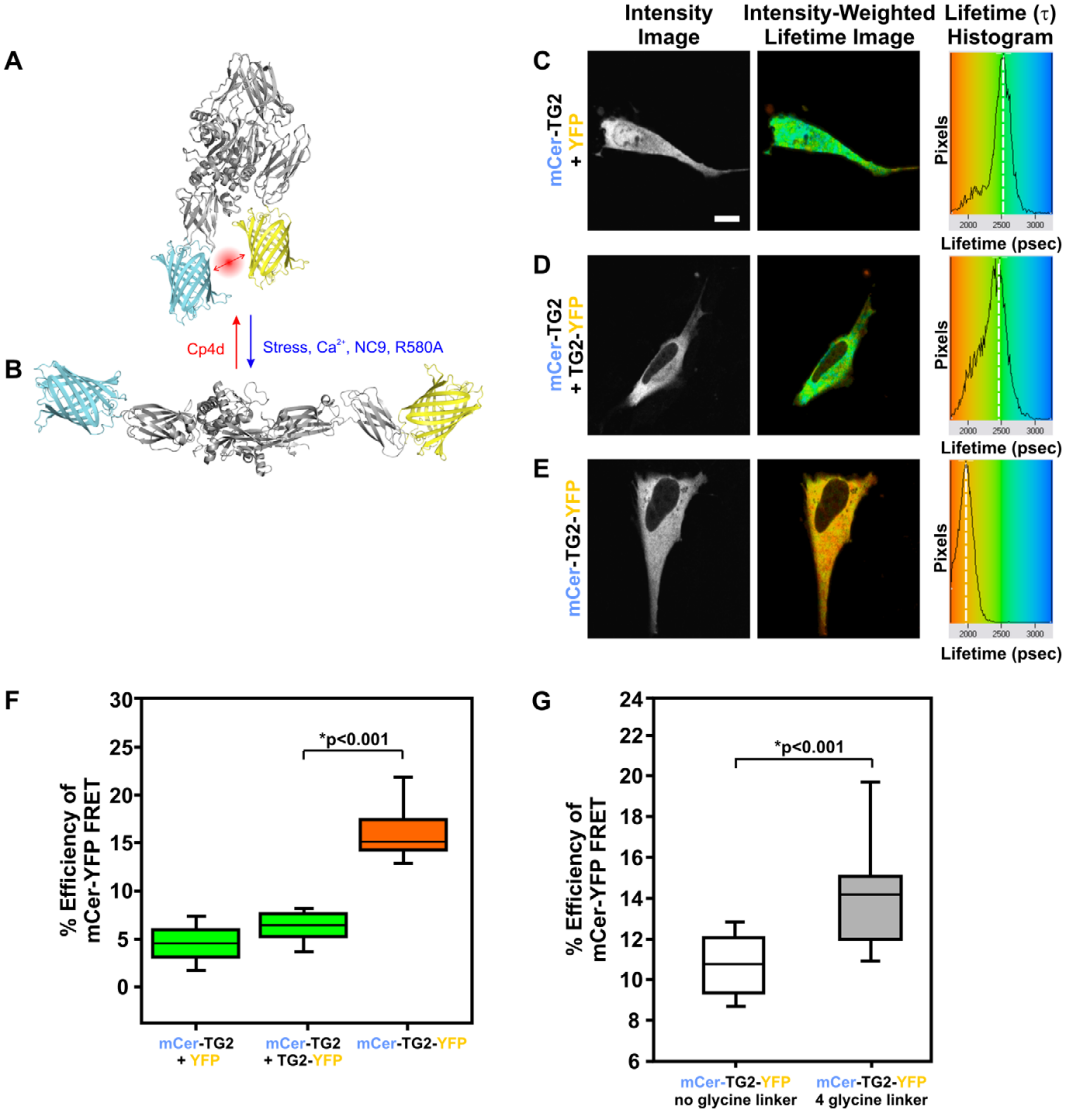
The Transglutaminase type 2 (TG2) Conformational FRET Sensor. (A and B) Speculative models of mCerulean-TG2-eYFP FRET sensor in a GDP/GTP bound closed conformation (A) and of mCerulean-TG2-eYFP sensor in a Ca^2+^ and substrate bound open conformation (B). (C) Sample FLIM image of mCerulean-TG2 with eYFP alone negative control. Intensity, intensity-weighted lifetime images and lifetime histograms are shown for every construct. Lifetimes shown in the intensity-weighted lifetime images are pseudo-coloured using a rainbow LUT that corresponds to the lifetime scale represented in the histogram. Dashed white lines within the histograms represents the approximate lifetime with the highest number of pixels. (D) Sample FLIM image of mCerulean-TG2 co-expressed with TG2-eYFP, a negative control for intermolecular FRET. (E) Sample FLIM image of mCerulean-TG2-eYFP conformational FRET sensor. (F) Quantitative FLIM-FRET data shown as percent efficiency of FRET for TG2 negative controls, as well as the mCerulean-TG2-eYFP FRET sensor. (G) Quantification of the effect on percent FRET efficiency of adding 4 glycines as linkers between the fluorophores and TG2. The boxes encompass the 25% to 75% confidence intervals, the line within indicates the data median and the whiskers represent the 5% to 95% confidence intervals. *p<0.001. N = 15 for 6 replicates. Scale bar represents 10 µm.

Using the molecular modelling software, PyMol [17], and previously published crystal structures of TG2 (PDB ID: 2Q3Z) [6], we measured the distances between the amino and carboxyl termini residues of TG2 in 3D space for both of its known conformations. The transition of TG2 from a closed to an open conformation shifts the distance between its termini from less than 10 nm to approximately 150 nm apart. We hypothesized that these distances would be amenable to detecting FRET and could be used to generate a conformational biosensor to analyze both the conformation and cellular localization of TG2 in live cells. We fused a donor mCerulean fluorescent protein and an acceptor yellow fluorescent protein (eYFP) fluorophore to the amino and carboxyl termini of TG2, respectively, and tested this construct under various conditions in live cells using TD-FLIM. Monomeric cerulean was chosen as a donor for FRET as this CFP variant has a mono-exponential lifetime decay and has significant spectral overlap with eYFP, making this pair optimal for FLIM-FRET [10]. As demonstrated by our model, when TG2 is bound to guanosine nucleotides in its closed conformation we would predict a robust increase in FRET efficiency, correlating with a decrease in the donor lifetime (Figure 1A). Alternatively, when a substrate molecule and/or calcium are bound to TG2 in the open conformation, the fluorophores are no longer in close spatial proximity and thus we would predict a reduction in FRET efficiency (Figure 1B).

Prior to testing our conformational sensor in live ST*Hdh* cells we determined that the mCerulean-TG2 fusion in the absence or presence of an acceptor (using eYFP alone, Figure 1C) had a lifetime of approximately 2.8 ns, consistent with the natural lifetime of mCerulean measured by others [10]. As predicted, we noted a significant decrease in donor lifetime (represented by the orange to red pseudo-colours in the photon-weighted lifetime image and a left shift in the lifetime histogram peak) with our mCerulean-TG2-eYFP sensor (Figure 1E and 1F). This can be interpreted as the TG2 population in the cell adopting a closed conformation. These results are consistent with the biochemical data which suggests that the majority of TG2 in cells under steady state conditions behaves as a GTPase/G-protein [7]. To establish that the observed decrease in lifetime was due to intramolecular FRET between individual TG2 molecules and not due to intermolecular FRET as an artefact of over-expression or protein aggregation, we co-expressed mCerulean-TG2 and TG2-eYFP constructs together on separate expression plasmids and saw no change in lifetime relative to the mCerulean-TG2+ eYFP alone control (Figure 1D and 1F). Despite the simplicity of this sensor, glycine linkers between the fluorophores and TG2 were tested in order to optimize the efficiency of energy transfer between the probes as well as increase the dynamic range for quantification (Figure 1G). We found that adding 4 glycines as linkers to both the amino and carboxyl termini provided the optimal FRET efficiency values. This was likely accomplished by less steric hindrance of TG2 protein dynamics by the fluorophores, as well as a better potential to align the fluorophore dipole moments in 3D space.

**Figure 2:**
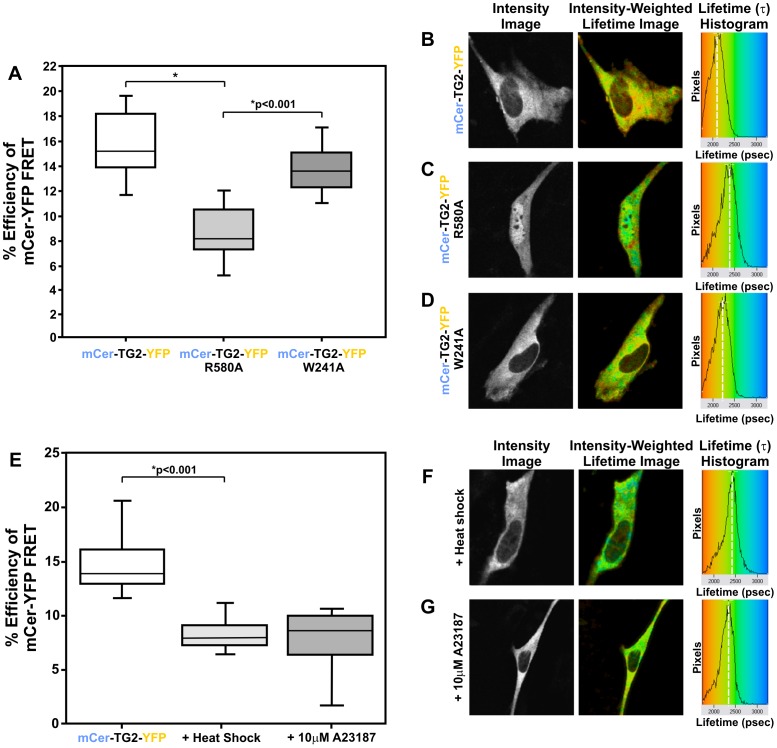
Point Mutations and Cell Stresses Affect TG2 Conformation in Live Cells. (A) Quantitative FLIM-FRET data shown as percent efficiency of FRET for the mCerulean-TG2-eYFP FRET sensor, GDP/GTP insensitive R580A mutant and catalytically inactive W241A mutant. Black line represents median values, boxes encompass 25% and 75% confidence intervals and whiskers indicate the 5% to 95% confidence intervals. *p<0.001. N = 15 for 4 replicates. (B–D) Representative FLIM images of data in (A). (E) Quantitative FLIM-FRET data shown as percent efficiency of FRET for the mCerulean-TG2-eYFP FRET sensor, or the sensor following either a 30 minute heat shock (42.5°C) treatment or a treatment with Ca^2+^ ionophore A23187 for 10 minutes. (F, G) Representative FLIM images of data in (E). *p<0.001. N>10 for 4 replicates. Scale bar represents 10 µm.

Next, we wanted to assay the effects of substitution mutations at critical residues on the conformation of TG2. Mutating arginine 580 of human TG2 to an alanine (R580A) abolishes all guanosine nucleotide binding [18], however, little is known regarding the effects of this mutation on the conformation of TG2. This R580A mutation causes a significant decrease in percent FRET efficiency (p<0.001) relative to the wild type (WT) TG2 biosensor (Figure 2A). This suggests that when TG2 cannot bind GDP/GTP, an increased amount of TG2 in the cell can be found in an open conformation. Tryptophan 241 is a conserved residue within human TG2 that is critical for its transamidating activity [19]. Mutating this residue to an alanine (W241A) abolishes its transamidating activity, while maintaining the ability of the enzyme to bind GDP/GTP [19]. As expected, this mutation had little effect on the conformation of the TG2 population relative to WT (Figure 2A). This is likely due to the ability of this TG2 mutant to still bind guanosine nucleotides.

To further validate this TG2 sensor, we tested the effect of various stresses on the conformation of TG2. Heat shock is a global cellular stress that activates many chaperones and signalling cascades involved in the heat shock response [20]. Heat shock causes a decrease in cellular ATP levels [21] and an increase in intracellular calcium levels [22], which we hypothesized would shift the intracellular population of TG2 towards an open conformation. Our data showed that a 30 minute heat shock treatment (at 42.5°C) causes a significant (p<0.001) decrease in the overall percent FRET efficiency within cells expressing the TG2 sensor (Figure 2B). All controls were also performed under the same conditions to ensure that this decrease in FRET efficiency was not due to an artefact of heat-denaturing the sensor fluorescent proteins. We also tested A23187 (calcimycin), an ionophore that makes the plasma membrane of cells permeable to divalent ions, rapidly increasing intracellular levels of calcium [23]. Treatment with the A23187 compound for 10 minutes had a similar effect on the conformation of TG2 as the heat shock treatment (Figure 2B), demonstrating that TG2 adopts an open conformation in live cells when exposed to high concentrations of calcium.

**Figure 3:**
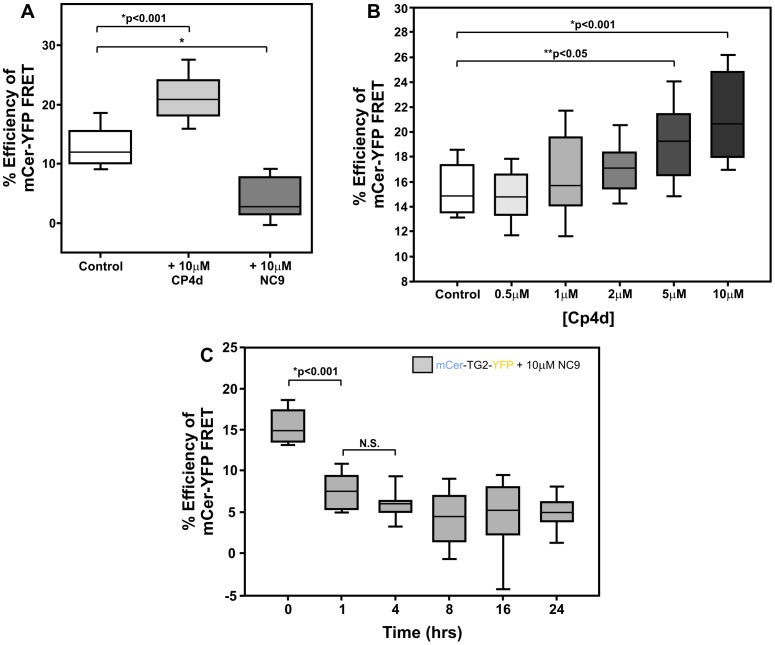
TG2 Inhibitors Differentially Affect TG2 Conformation. (A) Quantitative FLIM-FRET data shown as percent efficiency of FRET for the mCerulean-TG2-eYFP FRET sensor after a 16 hour treatment with either the irreversible TG2 inhibitor, NC9, or the reversible TG2 inhibitor, CP4d. (B) Percent efficiency of FRET graph generated following treatment of TG2 sensor with 5 increasing concentrations of CP4d. (C) Percent efficiency of FRET graph for TG2 sensor following treatment with 10 µM NC9 for 5 different time points from 0 to 24 hours. Black line represents median values, boxes encompass 25% and 75% confidence intervals and whiskers indicate the 5% to 95% confidence intervals. *p<0.001, **p<0.05 and N.S = not significant. N>10 for 4 replicates.

Lastly, we wanted to test the effects of various transamidation inhibitors on the conformation of TG2 to get more mechanistic insight into their mode of action *in vivo.* NC9 (α-N-carbobenzyloxy-γ-N-acryloyl-L-lysine(2-(2-dansylaminoethoxy)ethoxy)ethan-amide) is an irreversible inhibitor of the transglutaminase family that reacts with the active site of the enzyme and has a similar mode of action as the synthetic pentapeptide inhibitor used to generate the open conformation crystal structure of TG2 [6]. Therefore, NC9 has been hypothesized to stabilize the protein in an open conformation [24], [25]. To test this hypothesis, cells expressing the TG2 FRET sensor were treated with the NC9 compound for 16 hours. We measured a significant decrease in the percent FRET efficiency (Figure 3A and 3C; p<0.001) over non-treated cells. To test the conformational kinetics of this inhibition, we treated ST*Hdh* cells expressing the TG2 sensor with 10 µM NC9 and measured percent FRET efficiency temporally over a 24 hour time course (Figure 3C). We noted that NC9 elicited its most significant effects after 1 hour of treatment.

CP4d ((*E*)-1-(1-(4-nitrobenzyl)-1H-1,2,3-triazol-4-yl)-3-(4-nitrophenyl)prop-2-en-1-one) is a competitive, reversible and selective TG2 inhibitor with no known effect on the conformation of TG2 in cells [26]. In cells expressing the TG2 sensor, we noted that treatment with CP4d caused an increase in the percent FRET efficiency compared to no treatment, suggesting that this inhibitor maintains TG2 in the closed conformation to prevent transamidating activity. Treating cells with a range of CP4d concentrations ranging from 500 nM to 10 µM (Figure 3B) caused a progressive dose-response effect of this inhibitor on the conformation of TG2 with increasing concentrations of CP4d.

## Discussion

The various activities of TG2 have been directly implicated in the progression of celiac disease, arthrosclerosis, diabetes, cancer, glaucoma and the formation of cataracts [27].Additionally, TG2 has a role in multiple neurodegenerative diseases including Huntington’s (HD) [28], Parkinson’s (PD) as well as Alzheimer’s disease (AD) [29], [30].Studies of patient brains have shown that expression levels and transamidating activity of TG2 are highly elevated in these diseases, where it has been proposed that cell death may be caused by the aberrant cross-linking of substrates [30]. The role of TG2 in the cell death process is dependent on cell type, the stressor, its subcellular localization, its enzymatic role, and its conformation [24]. Using FLIM-FRET, the TG2 FRET biosensor not only provides a measure of the conformation and enzymatic role of TG2 but also provides resolution of the subcellular localization of TG2 in live cells. Therefore, this assay quickly provides several levels of information regarding TG2 and can be done under physiological conditions in living cells. Additionally, this TG2 sensor is a tool to determine if specific inhibitors are affecting both the conformation and/or localization of the enzyme under steady-state and cell stress conditions, and may be amenable to transgene expression in a mouse model.

While here we used FLIM to detect FRET changes, since both the donor and acceptor are on one molecule in a ratio of 1∶1, detection and quantification of FRET by the less costly methods amenable to high content or high throughput screening, i.e. sensitized emission [14] or acceptor photo-bleaching FRET [31]. It is also possible to image the temporal changes of FRET in live cells by the use of ratiometric imaging with SE-FRET.

Using this assay we were able to compare the effects of NC9 and Cp4d inhibitors both by dose-response and time-response in live cells expressing the TG2 FRET biosensor. As predicted by the original authors of the work describing NC9 [18], [25], this inhibitor was shown to force TG2 open and to stabilize it in this conformation [18]. Conversely, the Cp4d reversible TG2 inhibitor, was shown to promote the closed conformation of the TG2 biosensor [26]. This suggests that this inhibitor may still maintain GTP binding within the closed conformation, while completely inhibiting transamidation activity of TG2.

The differing activities of these TG2 inhibitors suggests that they may fall into three potential classes: Class I, which can promote a closed conformation of the enzyme while bound; class II, which can lock the enzyme in an open conformation; and a third class, class III, which does not have any conformational effects at all, yet can still inhibit the transamidation activity of TG2. Therefore, depending on the disease context, therapeutics targeted towards inhibiting the cross-linking activity of TG2 could be designed with the conformation of the enzyme in mind as to not affect the other potentially beneficial biological functions of TG2, such as calcium and/or GTP binding. Conversely, this biosensor could be used to screen new molecule inhibitors that specifically target the conformation of TG2, hence the calcium or GTP binding activities, if these are relevant to the disease pathology being studied.

## Materials and Methods

Ethics Statement: Full details of the study approval by The McMaster University Biosafety Committee.

Tissue Culture: Immortalized mouse striatal ST*Hdh* ^Q7/Q7^ cells were derived from normal WT mice and were grown as previously described (Atwal et al., 2007). Cells were a kind gift from Dr. Marcy Macdonald (MGH/Harvard) and previously characterized [32].

Cell Transfection: ST*Hdh* ^Q7/Q7^ cells were transfected using TurboFect *in vitro* transfection reagent (Fermentas) for 24–36 hours prior to imaging as previously described [28].

Plasmid Constructs: TG2 constructs were generated using PCR on human TG2 cDNA (generous gift from G.V.W. Johnson) with forward primer GATCAGATCTGGTGGCGGAGGGATGGCCGAGGAGCTGGTCTTAG with a BglII restriction site and reverse primer CTATGGTACCCCCTCCGCCACCGGCGGGGCCAATGATGACATTC with an Acc65I restriction site. TG2 R580A point mutation was introduced using inverse PCR on wild type TG2 with forward primer GCGGACCTCTACCTGGAGAATC and reverse primer CTCAGCCAGCAGGTAGCTGTTG. TG2 W241A mutation was introduced using inverse PCR on wild type TG2 with forward primer GCGGACAACAACTACGGG and reverse primer GCGTCCCAGCAGCACACC. All insert PCR products were cloned into a modified mCerulean-C1 plasmid with an eYFP insert cloned into BamHI and XbaI sites at the opposing end of the multiple cloning site (MCS).

TG2 Inhibitor Treatments: CP4d and NC9 inhibitor treatments were left on transfected ST*Hdh* ^Q7/Q7^ cells for ∼14–16 hours prior to use in FLIM-FRET experiments. TG2 inhibitors (NC9, CP4d) were confirmed to be active using an *in vitro* colorimetric transglutaminase assay (Sigma Cat # CS1070-1KT) at concentrations ranging from 500 nM to 15 µM. This assay was also used to confirm transglutaminase activity of the mCer-TG2-eYFP protein expressed in ST*Hdh* cells, which have little/no measureable endogenous TG2 activity.

Fluorescence Lifetime Imaging Microscopy (FLIM): FLIM was performed as previously described [28].

FLIM - ImageJ analysis: Using a Becker and Hickl FLIM plug-in for ImageJ (from www.macbiophotonics.ca), we imported both the tau and photon images for each cell to be quantified. The values on the tau (τ) images represent the fluorescent lifetimes of the donor fluorophore (mCerulean) at each pixel within the image, whereas the photon image represents the number of photons collected at each pixel. The values on the τ image were then set to only display a range from 1750–3250 picoseconds in order to minimize the contribution of background signal and auto-fluorescence in each image to the final quantifications. Using the freehand selection tool, each cell was then fully outlined on the photon image to reduce experimental bias, where this region of interest (ROI) was then applied to the τ image for measurement. Mean intensity measurements on the τ image represent the mean fluorescence lifetime at each R.O.I. FRET efficiency for each image was determined using the equation % FRET E = 1 − (average lifetime D.A/average lifetime D), where D.A lifetime indicates the average lifetime of mCerulean-TG2 fusions in the presence of the indicated acceptor and D lifetime indicates the overall average lifetime of mCerulean-TG2 fusions alone (no acceptor present). A minimum of 15 cells were quantified on each condition for every trial and subsequently graphed on a box-whisker plot. The box represents the 25% to 75% confidence intervals and the line within indicates the data median. The whiskers represent the 5% to 95% confidence intervals whereby outliers were removed if they fell outside 2 standard deviations from the mean.

Statistical Analysis: All statistical analysis was done using the SigmaPlot software 11.0 (Systat Software Inc.). For comparisons between two groups, Student’s T-tests were performed if data passed the normality assumptions. If data did not pass the normality test, it was analyzed by the Mann–Whitney method. For multiple pairwise comparisons, one-way analysis of variance (ANOVA) using the Student-Newman-Keuls method was performed if the data passed the normality test of distribution. If the data did not pass the normality assumptions, then we performed a one-way ANOVA on ranks using the Tukey test. For all FLIM quantifications, every cell was represented as its own N for that construct/treatment. Therefore, every box-whisker blot graph was generated using data from a single representative trial. Every construct/treatment trial was repeated a minimum of 3 times to ensure that trends were consistent between trials.
